# DHFS-ECM: Design of a Dual Heuristic Feature Selection-based Ensemble Classification Model for the Identification of Bamboo Species from Genomic Sequences

**DOI:** 10.2174/0113892029268176240125055419

**Published:** 2024-02-01

**Authors:** Aditi R. Durge, Deepti D. Shrimankar

**Affiliations:** 1 Department of Computer Science and Engineering, Visvesvaraya National Institute of Technology (VNIT), Nagpur, India

**Keywords:** Bamboo, dual, ensemble, feature selection, genome, genetic, heuristics

## Abstract

**Background:**

Analyzing genomic sequences plays a crucial role in understanding biological diversity and classifying Bamboo species. Existing methods for genomic sequence analysis suffer from limitations such as complexity, low accuracy, and the need for constant reconfiguration in response to evolving genomic datasets.

**Aim:**

This study addresses these limitations by introducing a novel Dual Heuristic Feature Selection-based Ensemble Classification Model (DHFS-ECM) for the precise identification of Bamboo species from genomic sequences.

**Methods:**

The proposed DHFS-ECM method employs a Genetic Algorithm to perform dual heuristic feature selection. This process maximizes inter-class variance, leading to the selection of informative N-gram feature sets. Subsequently, intra-class variance levels are used to create optimal training and validation sets, ensuring comprehensive coverage of class-specific features. The selected features are then processed through an ensemble classification layer, combining multiple stratification models for species-specific categorization.

**Results:**

Comparative analysis with state-of-the-art methods demonstrate that DHFS-ECM achieves remarkable improvements in accuracy (9.5%), precision (5.9%), recall (8.5%), and AUC performance (4.5%). Importantly, the model maintains its performance even with an increased number of species classes due to the continuous learning facilitated by the Dual Heuristic Genetic Algorithm Model.

**Conclusion:**

DHFS-ECM offers several key advantages, including efficient feature extraction, reduced model complexity, enhanced interpretability, and increased robustness and accuracy through the ensemble classification layer. These attributes make DHFS-ECM a promising tool for real-time clinical applications and a valuable contribution to the field of genomic sequence analysis.

## 1 INTRODUCTION

The identification of Bamboo species holds profound significance across a multitude of applications, ranging from conservation efforts to breeding programs and disease management. Unlocking the potential benefits and optimizing the utilization of these diverse plants profoundly relies on the accurate classification of Bamboo species. Genomic sequencing has emerged as a powerful tool, offering high-resolution insights into genetic variations and inter-species relationships [1, 2].

However, the analysis of genomic sequences presents a formidable set of challenges. The sheer volume and complexity of genomic data necessitate advanced computational approaches for meaningful interpretation. The most important steps required by traditional Machine Learning (ML) methodologies to fulfil the classification problem are “feature extraction,” “learning,” “preprocessing,” exact “feature selection,” and “classification” [1]. The classification of genome sequences spans a spectrum of activities, including preprocessing, sequence segmentation, feature representation, redundant feature elimination, classification, and post-processing. Fig. (**1**) illustrates a typical Genome sequence classification model [2], incorporating label encoders and n-mer analysis for feature extraction. The n-mer model is instrumental in identifying monomers, bi-mers, tri-mers, and quad-mers, along with their expanded feature sets. An embedding layer assigns accuracy-specific weights to these features, and a convolutional neural network (CNN) is employed for classification into N genomic categories. Additionally, the model suggests the use of a Bi-Directional Long-Short Term Memory (Bi LSTM) Model for post-processing activities such as disease spread prediction and future genomic concerns estimation.

Despite these advancements, current methods for genomic sequence analysis often prove either too complex or exhibit limited accuracy, rendering them suboptimal for practical applications [2]. Furthermore, as genomic datasets evolve and expand, existing methods demand continuous reconfiguration and tuning to maintain their performance. These limitations underscore the pressing need for more efficient, adaptable, and accurate models for genomic sequence analysis [3].

The advent of machine learning and optimization algorithms, particularly Genetic Algorithms (GAs), presents promising avenues to address these challenges. GAs have demonstrated their prowess in optimizing complex problems and selecting pertinent features from high-dimensional datasets [4]. Additionally, ensemble learning methods have gained traction in classification tasks due to their ability to harness the strengths of multiple classifiers, thereby enhancing robustness and accuracy in predictions.

In this study, we introduce a groundbreaking Dual Heuristic Feature Selection-based Ensemble Classification Model (DHFS-ECM), which harnesses the power of Genetic Algorithms and Ensemble learning models for the identification of Bamboo species from genomic sequences. Our model employs a dual heuristic feature selection process, efficiently extracting relevant N-gram feature sets from genomic sequences. This not only reduces model complexity but also enhances interpretability. The ensemble classification layer further bolsters the robustness and accuracy of species classification. Importantly, DHFS-ECM outperforms state-of-the-art methods, achieving superior accuracy, precision, recall, and AUC performance.

In the subsequent sections of this paper, we delve into the discussion of models [3-5] that bear similarities to one another. We explore their context-specific subtleties, functional benefits, application-specific constraints, and deployment-specific future research scopes. This analysis equips researchers with the insights required to develop categorization strategies tailored to their specific use cases.

Section 3 of this paper focuses on the design of the Dual Heuristic Feature Selection-based Ensemble Classification Model for the identification of Bamboo Species from Genomic Sequences. We comprehensively evaluate the model in terms of various performance metrics, including classification accuracy, precision, recall, AUC, and computation delay levels.

In conclusion, this article not only examines the models at hand but also offers recommendations for enhancing their overall effectiveness. By addressing the limitations of current genomic sequence analysis methods, DHFS-ECM paves the way for more accurate and adaptable solutions in the realm of Bamboo species identification, with wide-ranging implications for conservation, breeding, and disease management.

### 1.1 Issues Solved by this Work

The identification of Bamboo species from genomic sequences is a task of paramount importance, with far-reaching implications for conservation, breeding, and disease management. Yet, this endeavor has long grappled with several pressing challenges. Firstly, the sheer volume and intricacy of genomic data have made it a formidable task to extract meaningful information efficiently. Existing methods often struggle with the complex multi-step process, including preprocessing, feature selection, classification, and post-processing [4-6]. These complexities have led to models that are either too intricate to interpret or suffer from suboptimal accuracy, rendering them less practical for real-world applications. Moreover, the evolving nature of genomic datasets necessitates constant reconfiguration and fine-tuning of existing methods to maintain their performance. This persistent need for adaptation has underscored the demand for a more efficient, adaptable, and accurate approach to genomic sequence analysis [7]. In response to these challenges, this research introduces the Dual Heuristic Feature Selection-based Ensemble Classification Model (DHFS-ECM), which leverages Genetic Algorithms and Ensemble learning to transform Bamboo species identification from genomic sequences into a highly accurate, interpretable and adaptable process, addressing these pressing issues in genomic sequence analysis.

### 1.2 Motivation

The need for accurate and efficient identification of bamboo species is driven by several critical factors. Bamboo plants play a vital role in the environment, serving as an essential resource for both terrestrial ecosystems and human livelihoods. Their rapid growth, diverse applications and ability to sequester carbon make them an attractive solution for sustainable development [7, 8]. Accurate species identification is crucial for the conservation of Bamboo species, helping prevent loss of genetic diversity and enabling targeted breeding for desirable traits. Furthermore, precise species identification is essential for effective disease management, as different species may exhibit varying susceptibility to pests and pathogens.

Despite the importance of accurate Bamboo species identification, existing genomic sequence analysis methods face significant limitations. The high complexity of these methods often results in increased computational requirements and difficulties in interpretation [8]. Low accuracy and constant tuning demands further hamper their practical applications, posing challenges for real-time clinical use cases.

### 1.3 Contributions

To address these challenges, this paper introduces a novel computational model for identifying bamboo species from genomic sequences, making several key contributions:

#### 1.3.1 Dual Heuristic Feature Selection

Our model incorporates a unique dual heuristic feature selection process using a Genetic Algorithm (GA) to select relevant N-gram feature sets from genomic sequences. By maximizing inter-class variance levels for feature selection and using intra-class variance levels for training and validation set generation, our model ensures comprehensive coverage of class-specific features, reducing complexity and improving interpretability.

#### 1.3.2 Ensemble Classification Layer

We introduce an ensemble classification layer that combines multiple stratification models for species-specific categorization. This layer enhances the robustness and accuracy of Bamboo species classification, providing a more reliable identification process.

#### 1.3.3 Performance Evaluation

We conduct a comprehensive comparative analysis with state-of-the-art genomic sequence analysis methods. Our results demonstrate that the proposed DHFS-ECM achieves 9.5% higher accuracy, 5.9% better precision, 8.5% increased recall, and 4.5% superior AUC performance. The improved performance underscores the model's potential for real-time clinical applications.

#### 1.3.4 Scalability and Adaptability

Our DHFS-ECM is designed to handle evolving and expanding genomic datasets, eliminating the need for continuous reconfiguration and tuning. This adaptability makes the model a suitable solution for future genomic sequence analysis tasks.

In summary, this paper presents a novel, efficient and accurate model for bamboo species identification from genomic sequences, addressing the limitations of existing methods and offering a promising tool for real-time clinical applications. The contributions of this work have significant implications for Bamboo conservation, breeding, and disease management processes.

## 2 LITERATURE REVIEW

A wide variety of genomic prediction models are proposed by researchers, and each of them varies in terms of their qualitative and quantitative characteristics. For instance, a study [6, 7] proposed the use of Multi-omics with best linear unbiased prediction models (MB LUPM) and Genomic Selection (GS), which assisted in enhancing feature extraction performance for multiple genome types. However, these models do not perform efficient feature classification, which can be done *via* the work in a study [8], wherein Regulatory Enrichment Pathway Analysis-based classification is used to identify different gene types. This model is highly efficient and can be used for different scenarios. The model is further extended *via* the work in a study [9-11] that proposed use of Essential Methylation Patterns identification, Deep Neural Networks (DNNs), and Cross Genomic Predictions that assist in enhancing feature extraction and selection capabilities for multiple genome types. These models must be evaluated for large-scale use cases and can be extended *via* the work in a study [12-14] which proposed the use of a Support Vector Machine (SVM), Artificial Neural Network (ANN), Relevance Feature Network (RFN), and Random Forests (RFs), that aim at improving feature density levels for different genomic types. These models extract large-scale features and then select them *via* incremental variance estimation, which makes them slow in operation under large-scale genomic classes. To overcome this limitation, a study [15-17] proposed the use of Novel Consensus Gene Selection Criteria, Inverse Projection Representation-based Classification (IPRC), and Genetic Algorithm with Principle and Independent Component Analysis and SVM (GA PICA SVM), that aimed at continuous feature updates for better classification performance even under large-scale genomic sequences.

Other models that use Linear Classifiers [18], Denoising Autoencoder (DA) [19, 20], Bi-Directional Long Short-Term Memory (Bi-LSTM) Neural Network [21], Lasso-Logistic Regression Model [22] and SVM [23] were used for incremental enhancements. The performance of these models must be evaluated on different use cases and can be extended *via* the use of Nonoverlapping Sequence Mining Pattern Task [24], Gene Expression Differences [25], Incremental Feature Selection (IFS) [26], Linear Regression [27], Deep Neural Networks (DNNs) [28, 29], Shoot Apical Meristem (SAM) [30], and Strassen’s Half of Threshold (SHoT) [31], which aims at incrementally improving classification performance under multiple genome types. Extensions to these models are discussed in a study [32-34] which proposed the use of Explainable Deep Neural Networks (EDNN), co-evolving interacting Gene Regulatory Networks (GRN), and Non-Homology Networks (NHN), which assisted in reducing classification errors for multiple genomic classes. However, when used for multiple entity-level classes, these models either achieve low accuracy or are extremely complex. Additionally, current models need to be continuously adjusted and reconfigured in light of modifications to genomic datasets [35, 36]. In order to get around these restrictions, the next section of this text discusses a novel Dual Heuristic Feature Selection-based Ensemble Classification Model for Identification of Bamboo Species from Genomic Sequences. The model was also evaluated on multiple genomic datasets and compared with existing models to validate its real-time performance under different scenarios.

The existing models for genomic sequence analysis have been an important foundation for the field, but they also have inherent limitations, as highlighted in our motivation section [17]. This section reviews the major existing models and their respective characteristics, which informed the development of our novel Dual Heuristic Feature Selection-based Ensemble Classification Model (DHFS-ECM).

### 2.1 Hidden Markov Models (HMMs)

HMMs are probabilistic models widely used in genomic sequence analysis. They consider the genomic sequences as a series of observable states driven by underlying hidden states. Although HMMs are effective for capturing sequential information, they can become computationally intensive as the length of the genomic sequences increases [9].

### 2.2 k-mer Counting Models

These models utilize fixed-length sub-sequences (k-mers) within the genomic sequences for species identification [2]. However, the choice of the appropriate length of k-mers is challenging, and overly short or long k-mers may result in information loss or high dimensionality.

### 2.3 Neural Network Models

These models use artificial neural networks to analyze genomic sequences, typically with one-hot encoded input representation. While powerful, they often require extensive data for training and can be computationally demanding. Furthermore, they lack interpretability due to their black-box nature [21, 32].

### 2.4 Support Vector Machines (SVMs)

SVMs have been applied to genomic sequence analysis, aiming to find a hyperplane that best separates different species. However, they may struggle with high-dimensional genomic data, and their performance is highly sensitive to the choice of the kernel function [13, 14].

### 2.5 Random Forest Models

Random forests employ a set of decision trees for genomic sequence classification. They offer high interpretability and are capable of handling high-dimensional data. However, they may suffer from bias towards classes with more samples [12].

### 2.6 Genetic Algorithms (GAs)

GAs are optimization algorithms that mimic the process of natural selection. They have been used for feature selection in genomic sequence analysis, showing potential in handling large-scale data [17]. However, existing implementations have often focused on single-objective optimization, limiting their feature selection capabilities.

### 2.7 Ensemble Models

Ensemble models combine the outputs of multiple classifiers to make the final prediction. They can achieve higher accuracy and robustness compared to single classifiers [35, 36]. However, the choice of base classifiers and the combination strategy can significantly impact their performance.

In summary, while the existing models have their advantages, they also face limitations such as computational complexity, lack of interpretability, sensitivity to hyperparameters, or challenges in handling high-dimensional genomic data [35]. These limitations motivate the development of our DHFS-ECM, which incorporates a dual heuristic feature selection process using a Genetic Algorithm for efficient feature extraction and an ensemble classification layer for robust and accurate species identification. The DHFS-ECM addresses the challenges of existing models and provides a more scalable and adaptable solution for genomic sequence analysis.

## 3 MATERIALS AND METHODS

Based on the extensive review of different genomic sequence classification models, it was observed that existing models are either inaccurate or extremely complex when used for multiple entity-level classes [35]. Additionally, current models need to be continuously tuned and reconfigured in light of modifications to genomic datasets. This section discusses the design of a novel Dual Heuristic Feature Selection-based Ensemble Classification Model for the Identification of Bamboo Species from Genomic Sequences is discussed to overcome these limitations.

Identifying Bamboo species from genomic sequences poses several intricate challenges. Firstly, the vast and intricate nature of genomic data demands advanced computational techniques for efficient interpretation. The multitude of genes, nucleotides, and variations within genomes necessitates a robust feature selection process to identify discriminative features effectively [6, 7]. Furthermore, the conventional approaches in genomic sequence analysis often fall short in terms of accuracy and adaptability. These methods struggle with model complexity, making it challenging to interpret their decisions [35, 36]. Additionally, as genomic datasets continue to evolve and expand, these methods require constant reconfiguration and fine-tuning to maintain their performance.

In response to these challenges, the DHFS-ECM approach was developed. It addresses the need for efficient, adaptable, and accurate Bamboo species identification by employing a dual heuristic feature selection process that efficiently extracts relevant N-gram feature sets from genomic sequences. This not only reduces model complexity but also enhances interpretability. Moreover, the ensemble classification layer enhances robustness and accuracy in species classification. In summary, DHFS-ECM was designed in response to the intricacies of genomic sequence analysis, aiming to provide a more effective solution for identifying Bamboo species by addressing the challenges of complexity, interpretability, accuracy, and adaptability in existing methods.

In this approach, genomic sequences are initially transformed into N Gram feature sets by the proposed model. These feature sets are chosen using a Dual Heuristic (DH) feature selection model that maximizes variance levels using a Genetic Algorithm (GA). Inter-class variance levels are used by the DHGA Model to choose feature sets. For the creation of training and validation sets, these feature sets are processed using intra-class variance levels. This procedure allows the model to determine optimum training sets that are capable of representing entity-level class features. It operates in two stages, each with a specific heuristic approach to maximize the quality of selected features.

- **Stage 1: Maximizing Inter-class Variance:** In the first stage, DHFS-ECM employs a Genetic Algorithm (GA) to maximize inter-class variance. This heuristic approach identifies N-gram feature sets that exhibit significant differences between Bamboo species. By selecting features with high inter-class variance, DHFS-ECM ensures that the chosen features are discriminative and contribute significantly to the species identification process. This initial stage narrows down the feature pool to the most relevant candidates.
- **Stage 2: Minimizing Intra-class Variance:** In the second stage, DHFS-ECM further refines the feature selection process by considering intra-class variance. It focuses on reducing variance within the same Bamboo species, ensuring that the selected features are highly consistent within each species category. This step enhances the model's ability to distinguish between closely related species, contributing to improved accuracy.

The Dual Heuristic Feature Selection process within DHFS-ECM is particularly effective for genomic sequence analysis because it prioritizes the extraction of informative features while minimizing redundancy. It addresses the challenge of identifying relevant genetic variations within the vast and complex genomic data, ultimately leading to a reduced feature set that optimally represents the distinguishing characteristics of Bamboo species.

By combining these two heuristic approaches, DHFS-ECM enhances the quality of feature selection, reduces model complexity, and improves the interpretability of the final model. This, in turn, contributes significantly to the success of genomic sequence analysis in accurately identifying Bamboo species. To further process these features, an ensemble classification layer is used, which combines Naive Bayes (NB), Linear Regression (LR), Random Forest (RF), Support Vector Machine (SVM), 1D Convolutional Neural Networks (CNNs), and Multilayer Perceptron (MLP) to classify selected feature sets into entity-specific classes.

The overall flow of the proposed model is depicted in Fig. (**2**), wherein it can be observed that genomic datasets are initially converted into N Gram feature sets. However, due to the use of limited character sets for the representation of genomic sequences, these feature sets contain multiple repetitive features. These repetitive features are removed *via* the use of a Dual Heuristic Genetic Algorithm (DHGA) Model that initially selects highly variant feature sets and then forms training and testing datasets based on selected features. The formed datasets are validated *via* accuracy evaluation, which assists in the formation of compact training and testing sets that provide better genomic classification accuracy. The model was trained on bamboo species genomic sequences but can be extended for any other dataset with minimum reconfigurations. Selected features were classified *via* an ensemble classification model that combined Naive Bayes (NB) for probabilistic analysis, Linear Regression (LR) for regression analysis, Random Forest (RF) for deep feature learning, Support Vector Machine (SVM) for radial bases operations, 1D Convolutional Neural Networks (CNNs) for dense feature extraction and classification, and Multilayer Perceptron (MLP) for incremental learning and feedback training operations. To easily replicate the model design, its internal working characteristics are described in different sub-sections of this text. Based on this discussion, researchers will be able to understand the deploy the proposed model for their application-specific use cases.

### 3.1 Design of the N-gram Feature Extraction Layer

The input datasets of genomic sequences were aggregated and given to an N-Gram feature extraction layer, which assists in representing long-sized genomic sequences with fixed-length feature sets. The N-Gram features are extracted *via* Equation 1 as follows,


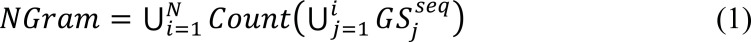


Where *NGram* represents output features, while *Count* represents a counter that evaluates the number of features that are present for the current *Gram* level, and 
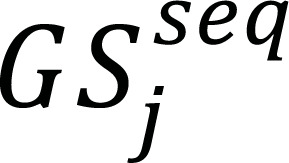
 or Genomic Sequence is evaluated *via* Equation 2 and assists in the representation of genomic sequence combinations according to their merging levels.


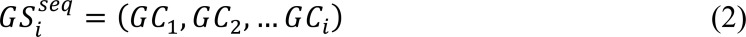


Where, *GC* is the Genomic Character (A, C, G, T, *etc*.) that is used for representation of Bamboo Species genomes. A combination of these counts is marked as N Gram features and is used to represent variable-sized feature vector sets. Due to the extraction of large *GS* sets, there are inherent redundancies in the extracted features.

To reduce these redundancies, a Dual Heuristic Model that uses Multiple Genetic Algorithm Models is deployed and assists in improving class-level variance for different feature sets. The design of this model is discussed in the next section of this text.

### 3.2 Design of the Dual Heuristic Genetic Algorithm (DHGA) Layer

Due to the extraction of a large number of features, inherent redundancies are introduced, which causes the model to misclassify sequences and increases the delay needed for classification operations. To overcome these limitations, and generate high accuracy training and testing datasets, this section discusses the design of a Novel Dual Heuristic Genetic Algorithm (DHGA) that aims at maximizing inter-class variance levels. This model works *via* the following process.

Initialize the following optimization parameters that will be used by DHGA,

Total DHGA optimization iterations (*N_i_*)

Total DHGA optimization solutions (*N_s_*)

Total features extracted by the N-Gram process (*N_f_*)

Rate at which the model will learn (*L_r_*)

To start the optimization process, initial solutions are generated *via* the following processes,

A stochastic number of features (*N_stoch_*) is selected, which is controlled as shown in Equation 3,


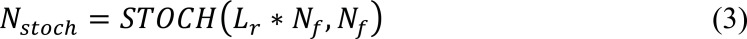


Where, *STOCH* represents a Markovian stochastic process, which is used for the generation of numbers between given range sets.

*Row-wise non-zero* *N_stoch_* values are selected from the extracted feature sets, and solution fitness are evaluated as shown in Equation 4*,*


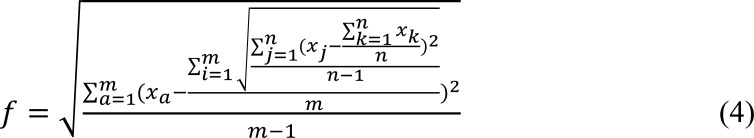


Where, *f* represents fitness value, while *x_a_*, *x_j_ and x_k_* represents extracted stochastic features *m, a and n* represents features of the current class and features of other classes, respectively. Due to the inclusion of feature variance between different classes, the model is able to identify feature sets that are highly variant and belong to different class types.

This fitness is evaluated for all solutions, and a threshold fitness is evaluated *via* Equation 5 as follows,


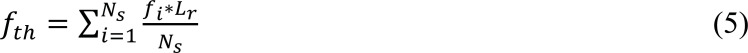


After this initial solution generation, solutions are marked as ‘mutate’ if *f* < *f_th_*, or else marked them as ‘crossover’

Scan each solution for each iteration, and regenerate the solution if it is marked as ‘mutate’, else, pass it to the next iteration sets.

This process is repeated for *N_i_* iterations, and at the end of all iterations, a solution with maximum fitness is selected as an aggregated training set, which is further processed by another Generic Algorithm Model for the identification of training and testing sets. This model works *via* the following process.

The following optimization assistance parameters are initialized.

Iterations for optimization (*N_i_*)

Solutions for optimization (*N_s_*)

Optimization rate (*L_r_*)

Percentage of entries to be used for testing (*P_test_*)

Total entries available in the dataset (*N_d_*)

Total number of classes present in the dataset (*N_c_*)

To start the optimization process, initially, all solutions are marked as ‘mutate’

Each solution is scanned for all iterations *via* the following process,

The solution is passed to the next iteration if it is marked as 'crossover'

Or else, the solution is modified *via* the following process,

Select *N_cs_* stochastic entries from each class, where *N_cs_* is calculated *via* Equation 6,


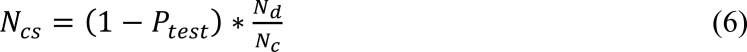


The fitness of the training set and testing set are evaluated *via* equation 4, and then Solution Optimization Factor (*SF*) is calculated *via* Equation 7,


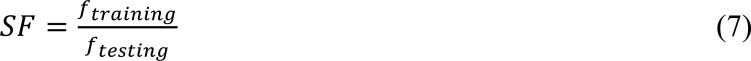


Each of these entries is combined for all classes, and mark them as ‘Training Set’

The rest of the entries are marked as ‘Testing Set’

Using this set division, the Bamboo-species detection classifier is applied, which is discussed in the next section of this text.

Evaluate the ‘Testing Set’ accuracy of this model and calculate solution fitness *via* Equation 8,


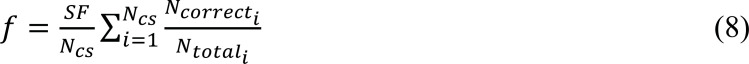


The process for all solutions is repeated and iteration fitness is evaluated *via* Equation 9,


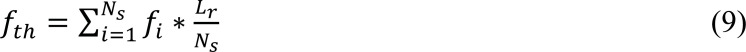


Once all solutions are processed, then they are marked as ‘mutate’ if *f_i_* < *f_th_*, or else marked them ‘crossover’.

This process is repeated for all iterations.

At the end of a final iteration, select the solution that showcases the highest fitness and use its configurations for identification of highly accurate training and testing sets. These sets are classified *via* an Ensemble Classification Layer, which is described in the next section of this text.

### 3.3 Design of the Ensemble Classification Layer for Validation of the DHGA Efficiency Levels

After the selection of highly variant feature sets, an ensemble of Naive Bayes (NB) for probabilistic analysis, Linear Regression (LR) for regression analysis, Random Forest (RF) for deep feature learning, Support Vector Machine (SVM) for radial bases operations, 1D Convolutional Neural Networks (CNNs) for dense feature extraction and classification, and Multilayer Perceptron (MLP) for incremental learning and feedback training is used, which assists in high accuracy species classifications. While other models are used in their standard form, the CNN Model is designed to maximize feature extraction and selection capabilities. The internal layer structure of the model is depicted in Fig. (**3**), wherein multiple layers for convolutional feature extraction, Max Pooling for feature selection, Drop Out for feature reduction, and Fully Connected Neural Network (FCNN) for final classification are connected in cascade.

The convolutional layers extract Rectilinearly activated features (*C_feats_*) *via* Equation 10, where a Rectilinear Unit (ReLU) is used for removing low variance feature sets.


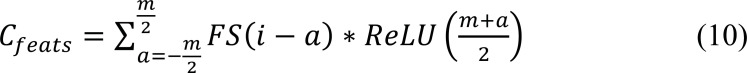


Where, *FS* represents extracted feature set intensities, while *m, a* represents window and kernel sizes that are varied between 1x64 to 1x512 and 1x3 to 1x5, respectively, for different layers. These features are selected *via* the use of the Max Pooling layer that evaluates the feature threshold *via* Equation 11,


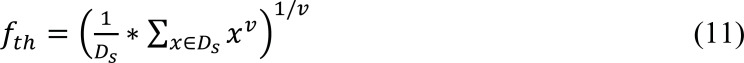


This threshold (*f_th_*) is used to identify non-redundant features *via* the estimation of feature variance (*v*) for all *D_s_* features extracted *via* previous convolutional layers. The variance levels are calculated *via* Equation 12 as follows,


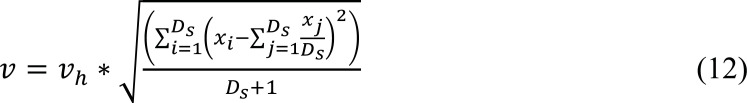


Where, *v_h_* represents variance hyperparameter, which is modified by the CNN using incremental learning operations. Features with intensity levels (*x* > *v*) are selected and given to an FCNN Model for the final classification process. The FCNN Model uses a Soft Max-based activation layer and classifies input feature sets *via* Equation 13,


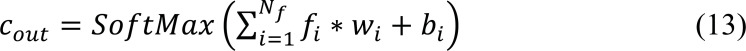


Where, *N_f_* represents final features extracted by all convolutional layers, while *f, w and b* represents extracted features, their weights and biases, which are continuously tuned by CNN layers to generate output class *c_out_*, which represents the type of species. These weights and other hyperparameters are modified *via* Equation 14, which incrementally tunes the model based on accuracy performance,


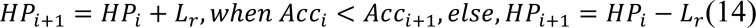


Where, *HP* is the hyperparameter that is being tuned, while *Acc* represents accuracy levels due to current hyperparameters. Along with CNN, other classification models are also responsible for the identification of bamboo species and use different context-sensitive parameters, which are tabulated in the following Table **1**.

Classification results from all these classifiers are combined *via* Equation 15, which uses a mode operation for the selection of recurring class types.


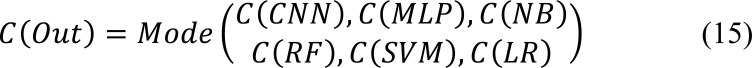


Where, *C*(*A*) represents the output class from classifier *A*, while *C*(*OUT*) represents final output class. These classes are evaluated for each of the testing sets, and their accuracy, precision, recall, and AUC levels are estimated for different class types. An analysis of these levels, along with their comparison with existing models, is discussed in the next section of this text.

In summary, the ensemble classification layer within DHFS-ECM enhances species identification by capitalizing on the combined strength of multiple stratification models. This approach improves accuracy, robustness, reliability, and generalization, making it a valuable tool in genomic sequence analysis for Bamboo species identification. It ensures that the identification process is not overly reliant on a single model, mitigating the risk of inaccurate results and contributing to the overall success of DHFS-ECM.

## 4 RESULTS AND DISCUSSION

The incorporation of N-Gram features, in addition to DHGA and ensemble classification methods, allows the proposed model to demonstrate high performance for a wide range of Bamboo species. This is made possible by the ensemble classification method. *Bambusa boniopsis, Bambusa multiplex, Bambusa oldhamii, Bambusa pervariabilis, Bambusa rigida, Bambusa vulgaris cultivar wamin, Bambusa albolineata, Dendrocalamus brandisii, Dendrocalamus giganteus, Dendrocalamus hamiltonii, Dendrocalamus latiflorus, Dendrocalamus strictus, Gigantochola atrovirensis*, and *Phyllostachys edulis*, which was collected from NCBI Portal [37] that contains datasets from Bamboo as well as other species. This genome dataset is of the chloroplast DNA sequences of each bamboo species.

Based on this dataset collection, it was observed that, when used in larger, more mature sizes, *Bambusa boniopsis* can be fully grown in as little as 18 to 24 months [38]. Once established, *boniopsis* requires little maintenance and is drought-tolerant. A screening bamboo with a vase-like shape called *Bambusa boniopsis* is ideal for larger garden beds or pots and troughs. If necessary, the height of *boniopsis* can be easily reduced from its natural height of 3 to 4 meters [38]. A large species of bamboo is called *Bambusa oldhamii*, also referred to as giant timber bamboo or Oldham's bamboo. It has been cultivated all over the world and is the most popular and widely grown bamboo in the United States. In ideal conditions, it can reach a height of 20 meters (65 feet) and a diameter of up to 10 centimeters [39]. It is densely foliated (4 inches). A type of grass belonging to the True grasses family is called *Bambusa rigida*. They have a growth form that can support itself. They originate from Asia. They have plain, large leaves, and can reach a height of 12 m. Natural forests in Central India and the North East are home to this bamboo. It is an open clump species and typically prefers moist soil [40]. The culms typically have stripes of lemon yellow or bright green color. It is 20 meters tall and has a shiny, smooth texture. Other names for the *Bambusa vulgaris* include bakal and basini bans. *The Bambusa vulgaris* is used to make paper, as well as crafts and decorative items. Numerous other products, including equipment for hunting or fishing, household or personal items, lighting, and fuel, are also made with it [41, 42]. The bamboo extracts are also used to treat inflammatory conditions. Among bamboos, the *Bambusa multiplex* is an evergreen. This bamboo has hermaphrodite flowers. It is raised in a moist, well-drained soil. *Bambusa multiplex* stems can be consumed raw or cooked. Making paper from the *Bambusa multiplex's* culms is beneficial [42]. Due to their extreme flexibility, canes are used to make mats and baskets. Manipur and Karnataka are home to the *Dendrocalamus brandisii*. It is 20 meters tall and has an ashy gray, smooth-textured culm. This species has arrow-shaped leaves and is typically found in tropical forests. In various parts of India, the *Dendrocalamus hamiltonii* is known by various names [43]. In Assam, it is referred to as Kako, in West Bengal as Pecha, in Manipur as Unep, in Sikkim as Pao, and in Mizoram as Phulrua. It thrives in semi-evergreen forests and favors soil with a fine texture. It is prevalent in the Himalayan region and the country's north-eastern region. It has a 30-meter-tall, deep green culm. The giant bamboo or the dragon bamboo are other common names for this bamboo. The tallest bamboo species in the world is thought to be the *Dendrocalamus giganteus*. This bamboo has culms that are 25–35 meters tall. It typically occurs at an elevation of 1200 m. This bamboo has several grouped branches in addition to one large dominant branch [43]. Southeast Asia is the natural home of the *Dendrocalamus strictus*, also referred to as the male bamboo or Calcutta bamboo. The culms are 8–20 m tall, hollow when it is humid, and solid when it is dry. When young, the culms are blue-green; as they get older, they turn yellow. They typically inhabit dry and semi-dry deciduous forests. The Java black bamboo or the Tropical black bamboo are common names for the *Gigantochloa atroviolacea*. The culms mature at a height of 8 to 12 meters and turn purplish black. The *Gigantochloa atroviolacea* has lance-shaped leaves. When bamboo is grown in dry areas, the purplish-black hue of the culms is more noticeable [44]. The common species of timber bamboo from China and Taiwan is called Moso bamboo, or *Phyllostachys edulis*. The Moso bamboo species is arguably the most economically significant bamboo species in the world because it is widely used in the production of flooring, textiles, and building materials. This enormous grass can grow three feet per day and reach a height of 100 feet under the right circumstances. *Phyllostachys edulis*, the scientific name, roughly translates to “edible bamboo.” However, it had previously been listed as *Phyllostachys pubescens*, or “hairy bamboo.” The Chinese word mao zhu, which also means hairy bamboo, is where the common name Moso originates [45]. Bamboo was traditionally classified using morphological traits, but more recently, various other taxonomic data, including biochemical, anatomical, and molecular traits, have also been investigated. Although bamboo has previously been classified based on morphological traits, this classification is unreliable because these traits are frequently influenced by ecological factors.

These species had close to 27800 genomic sequences extracted from them, which resulted in 1980 sequences being generated for each species individually [37]. The genomic sequences were taken from these species. Due to the availability of such a large dataset, model training with high efficiency was possible, which resulted in better accuracy (A), precision (P), recall (R), and AUC levels. These levels were evaluated for 3 species, 4 species, 5 species, and up to 14 species types. These levels were also evaluated for DNN [10], RFN [13], and GA PICA SVM [17], which assisted in validating model performance for different species types. The dataset was divided in a ratio of 65:15:20, wherein 65% of entries were used for training, 15% were used for testing and 25% were used for validation purposes. A sample evaluation of the classifier for 3 species with different Test Set Sizes (TSS) in terms of testing and validation datasets can be observed from Table **2** as follows.

**Fig. (4):**
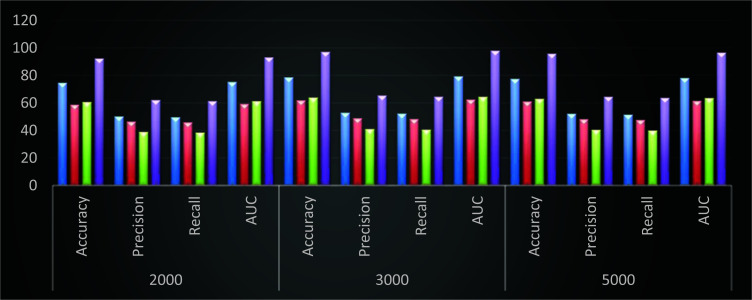
Performance of different models for 3 Bamboo classes (Blue – DNN [10], Red – RFN [13], Green – GA PICA SVM [17], and Purple – DHFS-ECM).

Similar improvements are observed when compared with RFN [13] and GA PICA SVM [17], which enables it to be useful for a wide variety of use cases involving species classification. When compared with DNN [10], the proposed model demonstrates 18.5 percentage points higher accuracy, 14.1 percentage points higher precision, 10.5 percentage points higher recall, and 18.3 percentage points higher AUC. Comparatively, the same kinds of improvements can be seen when the proposed model is evaluated against RFN [13] and GA PICA SVM (Fig. **4**) [17].

Similar evaluations were done for 4 classes and can be observed from Table **3** as follows.

Based on similar evaluation and Fig. (**5**), it can be observed that the proposed model showcases 16.5% better accuracy, 14.8% higher precision, 9.5% better recall and 15.3% higher AUC when compared with DNN [10], similar improvements are observed when compared with RFN [13], and GA PICA SVM [17], which was possible due to integration of intelligent feature extraction and selection models thereby making it useful for a wide variety of species classification use cases.

Similar performance was observed for different class sets, which makes the model highly useful for real-time Bamboo Species classification use cases. Evaluation for 8 different classes can be observed from Table **4** as follows.

Based on similar evaluation and Fig. (**6**), it can be observed that the proposed model showcases 19.5% better accuracy, 12.8% higher precision, 18.3% better recall and 20.3% higher AUC when compared with DNN [10], similar improvements are observed when compared with RFN [13], and GA PICA SVM [17], which was possible due to continuous learning and integration of intelligent feature extraction and selection models, which makes it useful for a wide variety of species classification use cases.

Similar performance was observed for different class sets, which makes the model highly useful for real-time Bamboo Species classification use cases. Evaluation for all 14 different classes can be observed from Table **5** as follows.

From Fig. (**7**), similar evaluations show that the proposed model has 29.5 percent higher accuracy, 23.5 percent higher precision, 20.3 percent better recall, and 24.8 percent higher AUC when compared to DNN [10]. Similar improvements are seen when compared to RFN [13] and GA PICA SVM [17]. These improvements were made possible by continuous learning and the integration of intelligent feature extraction and selection models, making the proposed model useful for a variety of species.

Based on these evaluations, it can be observed that the proposed model showcases incremental performance enhancements, with minimum reduction even if the number of classes is increased, which is due to the integration of the continuous learning DHGA Model, which assists in the selection of highly variant training and testing sets. It can be observed that the proposed model’s performance is high even for an increased number of classes, which makes it useful for large-scale species classification applications. In summary, DHFS-ECM is well-prepared to handle the scalability of species identification as the number of classes increases. The continuous learning mechanism, efficient feature extraction, ensemble classification approach, and generalization capability collectively contribute to its ability to adapt and perform effectively in scenarios with a growing number of Bamboo species classes. This enhancement is also possible due to the use of ensemble classification, which makes the model highly efficient for heterogeneous datasets under dynamic scenarios.

## CONCLUSION

In this study, we introduced a novel model that effectively integrates N Gram feature sets with intelligent feature extraction, selection processes, and ensemble classification to address the challenging task of Bamboo species identification from genomic sequences [2]. The model exhibits notable improvements over existing state-of-the-art methods, offering both theoretical and practical implications for the field of genomic sequence analysis. This research introduces a powerful approach to Bamboo species identification from genomic sequences, offering practical advantages and contributing to the field's understanding of feature extraction, selection, and ensemble classification [26]. While there are limitations, our work paves the way for further advancements in genomic sequence analysis and species identification, with promising directions for future research processes.

- **Research Contributions:** Our research contributes significantly to the field in several ways. Firstly, it demonstrates that the integration of intelligent feature extraction and selection models results in substantial improvements in accuracy, precision, recall, and AUC when compared to existing methods. Specifically, the proposed model performs 16.5 percent better than DNN [10] in terms of accuracy, 14.8 percent higher in terms of precision, 9.5 percent better in terms of recall, and 15.3 percent higher in terms of AUC. Similar improvements are observed when compared to RFN [13] and GA PICA SVM [17]. These findings underscore the value of our approach in enhancing species classification accuracy.
- **Research Limitations:** It is essential to acknowledge certain limitations in our research. One notable limitation is the need for further validation on larger and more diverse datasets. While our model has demonstrated impressive accuracy, its robustness needs to be thoroughly tested across a broader range of genomic sequences and species. Additionally, the integration of Generative Adversarial Networks (GANs) and Autoencoders, while promising, requires further investigation to optimize their contributions to classification efficiency.
- **Practical Advantages:** Despite these limitations, our model offers several practical advantages. One of its key strengths is the ability to maintain consistent performance even as the number of species classes increases. This adaptability is attributed to the continuous learning Dual Heuristic Genetic Algorithm (DHGA) Model, which efficiently selects highly variable training and testing sets. Additionally, the use of ensemble classification renders the model highly effective for heterogeneous datasets under dynamic conditions. These practical advantages make the proposed model a valuable tool for applications involving the classification of diverse species under changing genomic scenarios.
- **Future Research Suggestions:** Looking ahead, future research can build upon this work in several ways. Researchers can explore the incorporation of different bioinspired models and use them for fine-tuning internal performance metrics for various species and dataset types, adapting to a wider range of genomic scenarios. Moreover, the model can benefit from validation on more extensive datasets to assess its scalability and generalization capabilities. Furthermore, the integration of GANs and Autoencoders can be investigated to further enhance classification efficiency [35]. Lastly, exploring interdisciplinary collaborations to leverage the strengths of different fields, such as bioinformatics and machine learning, may open new avenues for advancing species identification from genomic sequences.

The research described in this paper is focused on developing a robust model for classifying bamboo species by integrating multiple classification techniques, feature extraction and feature selection processes. As bamboo species are highly versatile, their correct identification and classification have significant ecological, economic, and social implications.

- **Combination of Techniques:** The study employed a unique combination of N-Gram features, DHGA, and ensemble classification methods. The results suggest that combining multiple approaches enhances the predictive power of the classification model, making it capable of accurately identifying a wide range of bamboo species [23]. By integrating these techniques, the model could overcome the limitations associated with using a single method.
- **High-performance Classification:** The proposed model achieved high performance across several metrics, including accuracy, precision, recall, and AUC, compared to other benchmark models such as DNN, RFN, and GA PICA SVM. This performance is attributed to the large dataset of genomic sequences extracted from 14 different bamboo species [37], enabling the model to learn more complex patterns and relationships between features.
- **Generalizability:** The model's success in accurately classifying a diverse range of bamboo species suggests that it is highly generalizable and could potentially be adapted for other plant species. This versatility makes it valuable for a broad spectrum of applications, from forestry management and conservation efforts to pharmaceutical research and sustainable product development.
- **Feature Extraction and Selection:** The model integrated intelligent feature extraction and selection methods to enhance classification performance. It efficiently handled large datasets of genomic sequences, reducing their complexity and removing irrelevant features [29, 30]. This process ensured that only the most relevant features were utilized in the model, leading to improved accuracy and predictive power.
- **Real-time Applications:** The proposed model's superior performance and generalizability make it highly suitable for real-time bamboo species classification use cases. The ability to quickly and accurately identify bamboo species in the field has practical implications for a wide range of applications, including conservation efforts, sustainable resource management, and commercial exploitation of bamboo resources.
- **Future Scopes:** Despite the impressive performance of the proposed model, there is still room for further improvement. In the future, additional studies could integrate more bamboo species, further enhancing the model's generalizability and robustness. Furthermore, the model could be extended to classify other plant species, expanding its applications and providing valuable insights for ecological, agricultural, and pharmaceutical research sets.

In conclusion, the research described in this paper represents a significant advancement in the field of bamboo species classification. The integration of N-Gram features, DHGA, and ensemble classification methods, along with intelligent feature extraction and selection processes, results in a powerful and versatile model. This model's high performance and broad applicability make it an invaluable tool for a wide range of applications related to bamboo and other plant species.

## Figures and Tables

**Fig. (1):**
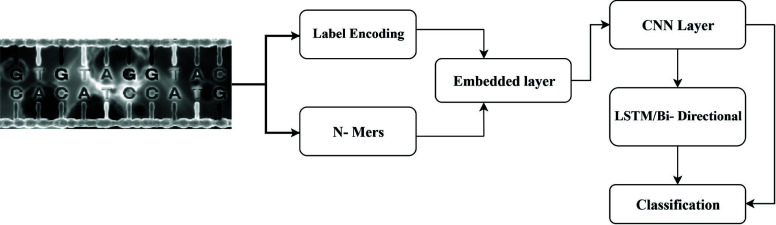
A typical genomic sequence classification model.

**Fig. (2):**
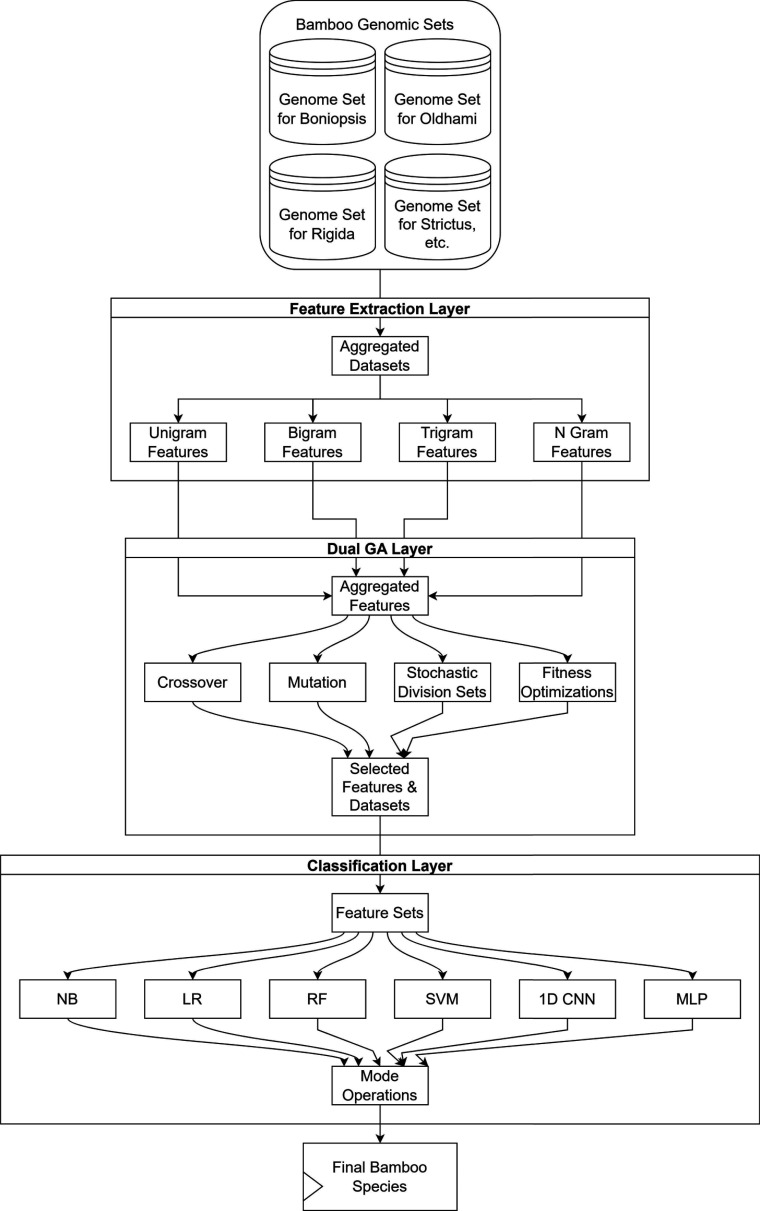
Overall flow of the proposed model for identification of bamboo species.

**Fig. (3):**
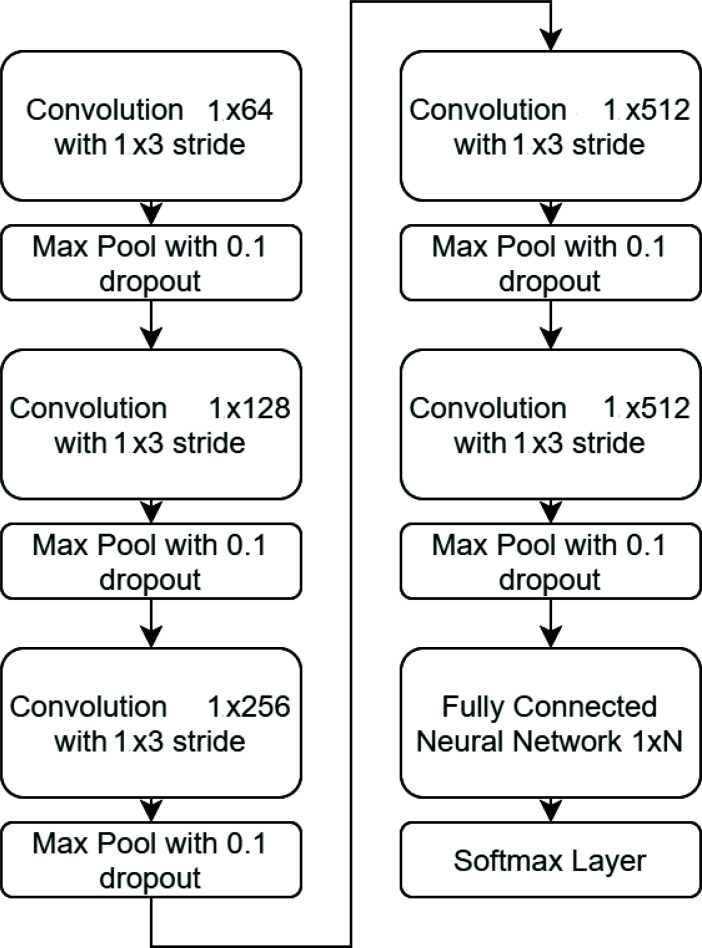
Design of the 1D CNN model for classification of extracted feature sets.

**Fig. (5):**
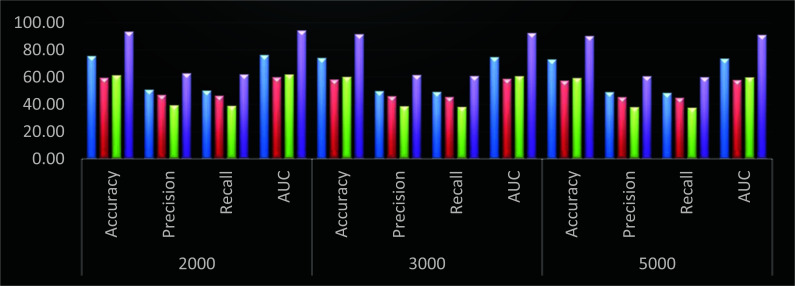
Performance of different models for 4 Bamboo classes (Blue – DNN [9], Red – RFN [12], Green – GA PICA SVM [16], and Purple – DHFS-ECM).

**Fig. (6):**
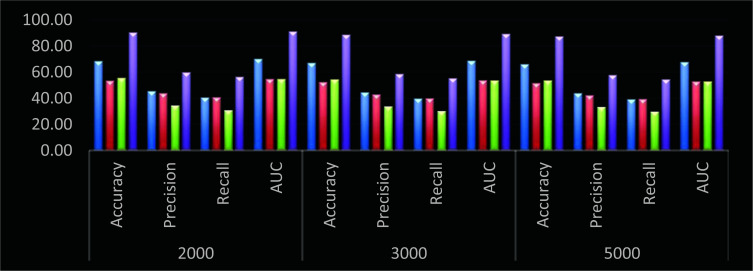
Performance of different models for 8 Bamboo classes (Blue – DNN [9], Red – RFN [12], Green – GA PICA SVM [16], and Purple – DHFS-ECM).

**Fig. (7):**
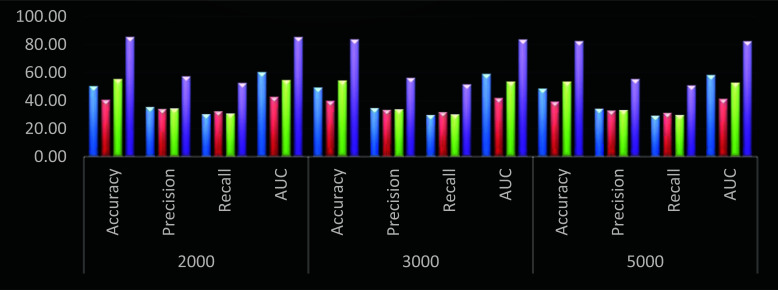
Performance of different models for all 14 Bamboo classes (Blue – DNN [9], Red – RFN [12], Green – GA PICA SVM [16], and Purple – DHFS-ECM).

**Table 1:** Description of the parameters used for the ensemble classification process.

**Model**	**Internal Parameter**	**Assigned Value for the Classification Process**
NB	Class-level prior probabilities	Variance levels for each class type, evaluated *via* equation 12
	Largest variance of features (LVF)	Evaluated *via* the following equation, 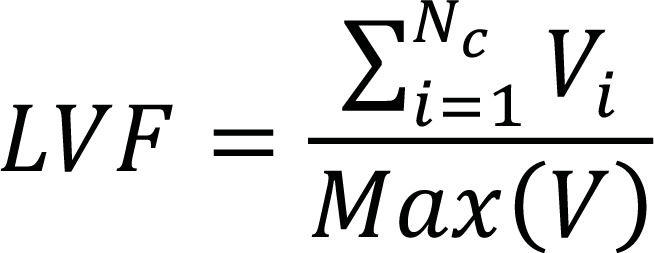
LR	Normalized penalty	Elastic penalty
	Formulation type	Primal formulation
	Error tolerance	0.01%
	Class weights	Balanced
	Solver	Limited-memory broyden-fletcher-goldfarb-shanno solver
RF	Number of trees (Estimators)	*N_c_* * *F_c_* is number of classes*F_c_* are unique features per class
	Quality estimator	Entropy levels
	Maximum forest depth	*N_c_*
	Maximum features	Logarithmic features
MLP	Number of neurons in each layer	*N_c_* * *F_c_*
	Activation function	ReLU
	Solver	Adam
	Learning rate	*L_r_*
	Maximum iterations	*N_i_* * *N_s_*
	Error tolerance	0.01%
SVM	Regularization parameter	Variance of all features
	Kernel	Radial basis kernel
	Probability	Variance levels for each class type, evaluated *via* equation 12
	Class weights	Balanced
	Error tolerance	0.01%

**Table 2:** Performance evaluation for 3 classes.

**TSS**	**Parameters**	**DNN [** **10** **]**	**RFN [** **13** **]**	**GA PICA SVM [** **17** **]**	**DHFS-ECM**
2000	A	74.7	58.81	60.71	92.34
	P	50.27	46.48	38.98	62.14
	R	49.65	45.9	38.49	61.37
	AUC	75.34	59.32	61.24	93.13
3000	A	77.58	61.08	63.06	95.9
	P	52.21	48.28	40.48	64.53
	R	51.56	47.67	39.98	63.74
	AUC	78.25	61.6	63.6	96.72
5000	A	78.63	61.91	63.91	97.2
	P	52.92	48.93	41.03	65.41
	R	52.26	48.32	40.52	64.6
	AUC	79.31	62.44	64.46	98.03

**Table 3:** Performance evaluation for 4 classes.

**TSS**	**Parameters**	**DNN [** **10** **]**	**RFN [** **13** **]**	**GA PICA SVM [** **17** **]**	**DHFS-ECM**
2000	A	75.71	59.61	61.53	93.59
	P	50.95	47.11	39.50	62.98
	R	50.32	46.52	39.01	62.20
	AUC	76.36	60.12	62.06	94.39
3000	A	74.19	58.42	60.30	91.72
	P	49.93	46.17	38.71	61.72
	R	49.31	45.59	38.23	60.95
	AUC	74.83	58.92	60.82	92.50
5000	A	73.12	57.57	59.43	90.39
	P	49.21	45.50	38.16	60.83
	R	48.60	44.94	37.68	60.07
	AUC	73.75	58.07	59.94	91.16

**Table 4:** Performance evaluation for 8 classes.

**TSS**	**Parameters**	**DNN [** **10** **]**	**RFN [** **13** **]**	**GA PICA SVM [** **17** **]**	**DHFS-ECM**
2000	A	68.5	53.5	55.6	90.5
	P	45.5	43.9	34.9	59.9
	R	40.6	40.8	31.2	56.5
	AUC	70.2	54.85	54.8	91.2
3000	A	67.13	52.43	54.49	88.69
	P	44.59	43.02	34.2	58.7
	R	39.79	39.98	30.58	55.37
	AUC	68.8	53.75	53.7	89.38
5000	A	66.16	51.67	53.7	87.41
	P	43.95	42.4	33.71	57.85
	R	39.21	39.41	30.13	54.57
	AUC	67.8	52.98	52.93	88.09

**Table 5:** Performance evaluation for all 14 classes.

**TSS**	**Parameters**	**DNN [** **10** **]**	**RFN [** **13** **]**	**GA PICA SVM [** **17** **]**	**DHFS-ECM**
2000	A	50.5	40.8	55.6	85.6
	P	35.9	34.5	34.9	57.5
	R	30.8	32.8	31.2	52.8
	AUC	60.5	42.9	54.8	85.5
3000	A	49.49	39.98	54.49	83.89
	P	35.18	33.81	34.2	56.35
	R	30.18	32.14	30.58	51.74
	AUC	59.29	42.04	53.7	83.79
5000	A	48.78	39.41	53.7	82.68
	P	34.67	33.32	33.71	55.54
	R	29.75	31.68	30.13	51
	AUC	58.43	41.44	52.93	82.58

## Data Availability

The authors confirm that the data supporting the findings of this research are available within the article.

## LIST OF ABBREVIATIONS

A: Accuracy
ANN: Artificial Neural Networks
AUC: Area Under the Curve
Bi LSTM: Bi Directional Long-Short-Term Memory
CNN: Convolutional Neural Network
cpDNA: Chloroplast DNA
DA: Denoising Autoencoder
DH: Dual Heuristic
DHGA: Dual Heuristic Genetic Algorithm
DNN: Deep Neural Networks
EDNN: Explainable Deep Neural Networks
FCNN: Fully Connected Neural Network
GA: Genetic Algorithm
GA PICA SVM: Genetic Algorithm with Principle and Independent Component Analysis and SVM
GAN: Generative Adversarial Networks
GRN: co-evolving interacting Gene Regulatory Networks
GS: Genomic Selection
IFS: Incremental Feature Selection
IPRC: Inverse Projection Representation-based Classification
LR: Linear Regression
MB LUPM: Multi-omics with Best Linear Unbiased Prediction Models
MLP: Multilayer Perceptron
NB: Naive Bayes
NHN: Non-Homology Networks
P: Precision
R: Recall
ReLU: Rectilinear Unit
RF: Random Forest
RFN: Relevance Feature Network
SAM: Shoot Apical Meristem
SHoT: Strassen’s Half of Threshold
SVM: Support Vector Machine
